# Assessment of Artificial Intelligence Strategies to Estimate the Strength of Geopolymer Composites and Influence of Input Parameters

**DOI:** 10.3390/polym14122509

**Published:** 2022-06-20

**Authors:** Kaffayatullah Khan, Waqas Ahmad, Muhammad Nasir Amin, Ayaz Ahmad, Sohaib Nazar, Majdi Adel Al-Faiad

**Affiliations:** 1 Department of Civil and Environmental Engineering, College of Engineering, King Faisal University, Al-Ahsa 31982, Saudi Arabia; mgadir@kfu.edu.sa; 2 Department of Civil Engineering, COMSATS University Islamabad, Abbottabad 22060, Pakistan; waqasahmad@cuiatd.edu.pk (W.A.); sohaibnazar@cuiatd.edu.pk (S.N.); 3 MaREI Centre, Ryan Institute, School of Engineering, College of Science and Engineering, National University of Ireland Galway, H91 HX31 Galway, Ireland; a.ahmad8@nuigalway.ie; 4 Department of Chemical Engineering, College of Engineering, King Faisal University, Al-Ahsa 31982, Saudi Arabia; malfaiad@kfu.edu.sa

**Keywords:** alternative binder, geopolymers, compressive strength, artificial intelligence, machine learning, modeling, SHAP plots

## Abstract

Geopolymers might be the superlative alternative to conventional cement because it is produced from aluminosilicate-rich waste sources to eliminate the issues associated with its manufacture and use. Geopolymer composites (GPCs) are gaining popularity, and their research is expanding. However, casting, curing, and testing specimens requires significant effort, price, and time. For research to be efficient, it is essential to apply novel approaches to the said objective. In this study, compressive strength (CS) of GPCs was anticipated using machine learning (ML) approaches, i.e., one single method (support vector machine (SVM)) and two ensembled algorithms (gradient boosting (GB) and extreme gradient boosting (XGB)). All models’ validity and comparability were tested using the coefficient of determination (R2), statistical tests, and k-fold analysis. In addition, a model-independent post hoc approach known as SHapley Additive exPlanations (SHAP) was employed to investigate the impact of input factors on the CS of GPCs. In predicting the CS of GPCs, it was observed that ensembled ML strategies performed better than the single ML technique. The R2 for the SVM, GB, and XGB models were 0.98, 0.97, and 0.93, respectively. The lowered error values of the models, including mean absolute and root mean square errors, further verified the enhanced precision of the ensembled ML approaches. The SHAP analysis revealed a stronger positive correlation between GGBS and GPC′s CS. The effects of NaOH molarity, NaOH, and Na_2_SiO_3_ were also observed as more positive. Fly ash and gravel size: 10/20 mm have both beneficial and negative impacts on the GPC′s CS. Raising the concentration of these ingredients enhances the CS, whereas increasing the concentration of GPC reduces it. Gravel size: 4/10 mm has less favorable and more negative effects. ML techniques will benefit the construction sector by offering rapid and cost-efficient solutions for assessing material characteristics.

## 1. Introduction

Ordinary Portland cement (OPC), the extensively utilized cementitious ingredient in concrete globally, is connected with high energy demand and significant CO_2_ discharges as a result of the manufacturing processes [1,2,3,4,5,6]. OPC manufacturing emits around 4 billion tons of CO_2_ annually and emits nearly 5–7% of overall CO_2_ worldwide [7,8]. Numerous measures have been attempted to mitigate the effects of OPC production and consumption in response to growing concerns about environmental protection and climate change [9,10,11,12,13]. These consist of the use of supplementary cementitious materials (SCMs), as well as the recycling of waste materials, in order to decrease the dependency on OPC in construction [14,15,16,17,18,19,20]. However, the percentages of these SCMs utilized in place of OPC are frequently regulated [21,22,23]. Using fly ash as an example, while it exhibits pozzolanic properties at various phases of OPC hydration, it plays a minor influence in early strength development [24,25,26,27,28,29]. The inclusion of fly ash can decrease the rate of initial hydration and lengthen the time of setting [30,31,32], limiting their application in large contents. Using alkali activation to create more ecologically friendly cementitious binders is one of the most researched ways of completely replacing OPC [33,34,35,36]. Alkali-activated materials (AAMs) do not require the elevated-temperature, high-energy calcination method used to produce OPC clinker [37,38,39]. AAMs, also known as geopolymers, are polymeric aluminosilicate cementing ingredients with three-dimensional spatial complex topologies that are mostly composed of industrial wastes (e.g., fly ash) and activated with an alkaline agent (e.g., NaOH, Na_2_SiO_3_) [40,41,42]. Due to their unique chemical composition, geopolymers have advantageous mechanical performance and durability. From environmental aspects, these methods are more appealing than OPC-based mixes due to the reuse of waste materials as the primary binder component [43,44,45].

Predictive models for material strength are being developed to eliminate needless experimental repeats and ingredient wastages. Numerous common models are used to simulate the properties of concrete, including best-fit curves (established on regression evaluation). Nevertheless, due to the nonlinear character of cementitious materials [46,47], regression techniques created in this manner might not sufficiently reflect the material′s basic behavior. Furthermore, regression approaches may overestimate the contribution of certain elements [48]. Artificial intelligence methods, such as supervised machine learning (ML), are among the most refined modeling methods used in the present study area [49,50,51,52,53]. These methods utilize input variables to model responses, and the yield models are supported by testing. ML techniques are used to anticipate concrete and bituminous mixtures characteristics [54,55,56,57,58,59,60,61,62]. Most previous ML-based studies have concentrated on predicting the compressive strength (CS) of OPC-based materials [63,64,65,66,67,68,69]; only a few articles concentrated on predicting the characteristics of geopolymer composites.

This study focuses on the application of ML techniques to assess the CS of GPCs. Three distinct ML techniques were employed: support vector machine (SVM), gradient boosting (GB), and extreme gradient boosting (XGB), and their performance was evaluated using coefficients of determination (R^2^), k-fold, statistical tests, and divergence of estimated results (errors) from those of experimental. SVM is a single ML method, while GB and XGB are ensemble ML methods [70]. This study is noteworthy because it predicts the CS of GPC by applying both single and ensemble ML algorithms. However, experimental researches require considerable human effort, experimental expenses, and time for ingredients collection, casting, curing, and testing. By addressing the aforementioned challenges through the use of new methods such as ML, the building industry will obtain an advantage. Due to the fact that a range of elements, such as precursors, activators, aggregates, etc., affect GPC’s CS, it is hard to investigate their collective effect using experimental methods. With minimum effort, ML methods are adept at determining the collective impact of their components in a single study. A data set is required for ML methods, which might be acquired from the literature since various experimental studies have been undertaken to establish the CS of GPC. The collected data may subsequently be utilized to train ML algorithms and forecast material attributes. Previous research used ML algorithms to predict the GPC′s mechanical strength using a reduced quantity of input factors and data samples. For instance, Dao et al. [71] used ML methods to predict the CS of GPC using three input factors and 210 data points. Similar to the previous study, another employed four input factors and 210 data samples [72]. The current research employed nine input factors and 371 data samples to anticipate the CS of GPCs and evaluate the effectiveness of several ML approaches. It is believed that increasing the number of input factors and data samples would improve the accuracy of ML approaches. The purpose of this work is to discover the best suitable ML technique for predicting the CS of GPC and the effect of several parameters on GPC strength.

This study is also novel in that it involves the comparative study of the employed ML algorithms for recommending the high-precision approach in the further studies for predicting the CS of GPC. These approaches are also beneficial for the researchers and the construction industries to minimize the experimental efforts, cost, and time of the project.

## 2. Methods

### 2.1. Data Retrieval and Analysis

**Figure 1:**
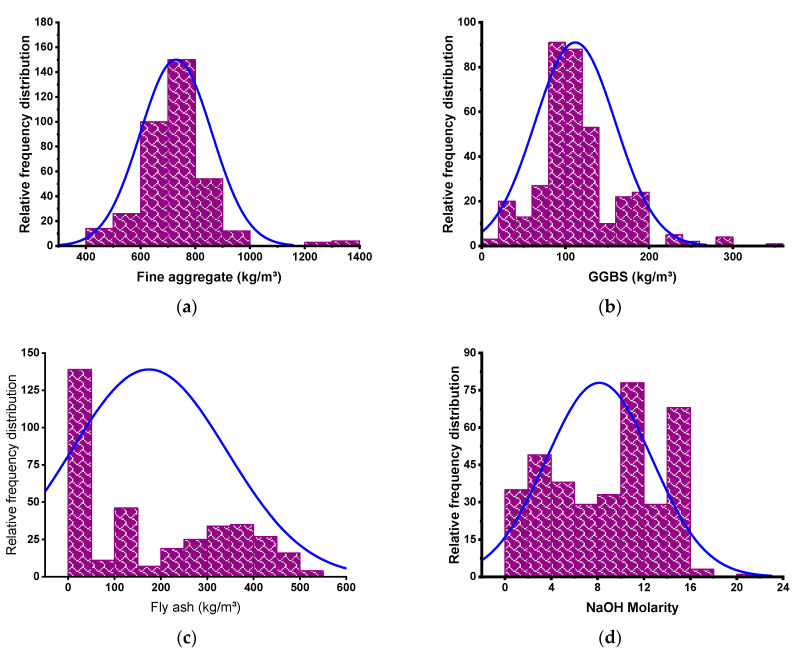

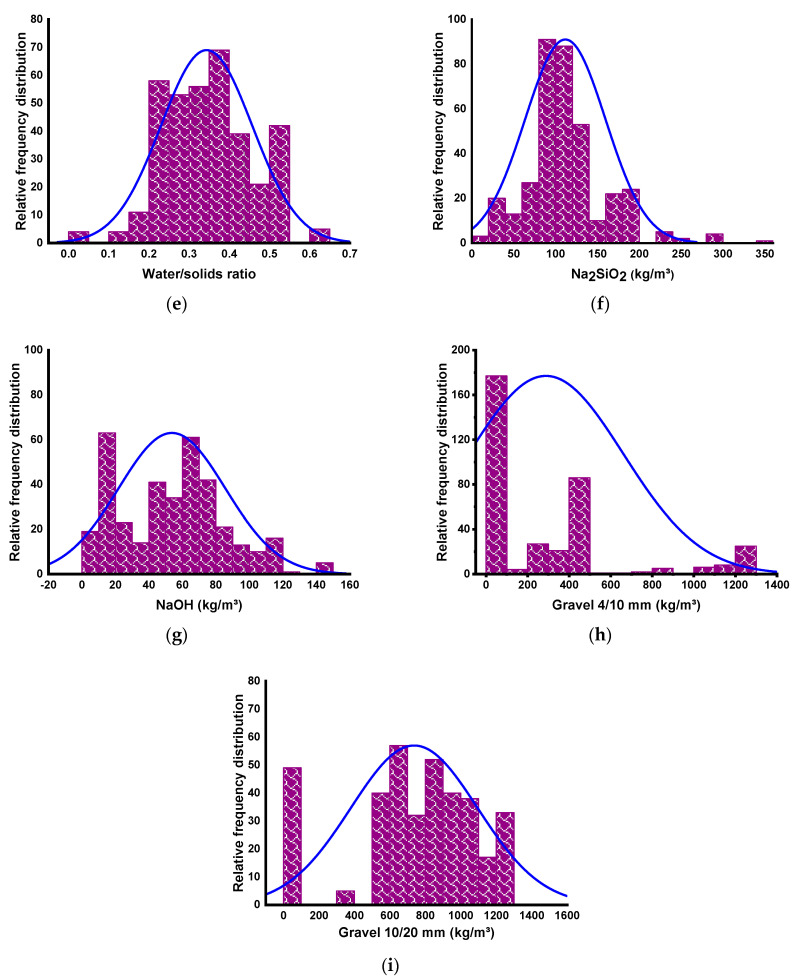
Relative frequency dispersal of inputs. (**a**) Fine aggregate; (**b**) GGBS; (**c**) Fly ash; (**d**) NaOH molarity; (**e**) Water/solids ratio; (**f**) Na_2_SiO_2_; (**g**) NaOH; (**h**) Gravel 4/10 mm; (**i**) Gravel 10/20 mm.

**Figure 2:**
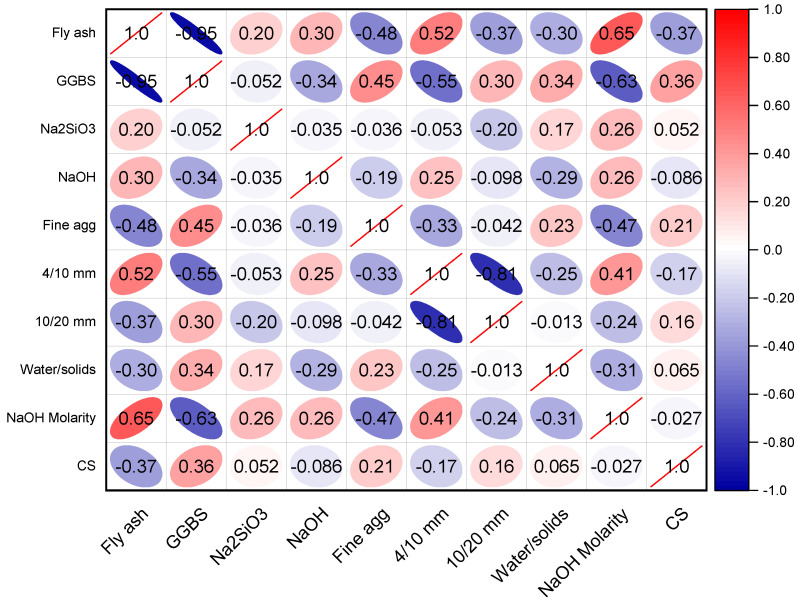
Correlation of input parameters with compressive strength.

SML methods require a wide variety of input variables in order to produce the desired output [73]. The CS of GPC was calculated using data from the scientific literature (see Table S1 in supplementary materials). In order to prevent bias, experimental data were selected at random from the published literature. This study gathered CS-based data points to run the algorithms, whereas the majority of articles studied additional aspects of GPC. Fine aggregate, GGBS, fly ash, NaOH molarity, NaOH, water/solids ratio, Na_2_SiO_3_, and gravel size: 10/20 and 4/10 mm were included as input variables in the algorithms, with CS serving as the output parameter. The number of inputs and datasets has a major effect on the output of the model [41]. In the present study, 371 data points were utilized to execute ML algorithms (see Supplementary Materials). The data were retrieved by keeping the mix proportions and required outcome in consideration as models required a similar number of input parameters for each mix to run it for the required output. The data used in this study were retrieved from the literature, so many of the tests were performed in the different zones, testing setups, and geometry setups. However, this variation in different testing setups, arrangements, or geometry of samples does not affect the study’s main findings, as the models required only input variables and outcomes, irrespective of the testing setup and arrangements. Each input variable′s descriptive statistics are summarized in Table 1. The normalization process was also adopted for the selected data. Normalization is the process of structuring data in a database. This entails constructing tables and developing links between those tables according to rules designed both to safeguard the data and to make the database more adaptable by removing redundancy and inconsistent reliance. The word “descriptive statistics” refers to a collection of short, factual measures that yield an outcome, which may be the entire population or a subset of it. The mean, median, and mode variables show fundamental tendencies, whereas the maximum, minimum, and standard deviation variables represent variation. Table 1 contains all the mathematical terms for the input variables of the model. The distribution of each input factor with CS is depicted in Figure 1. The frequency distribution is shown diagonally, along with the correlation between each input and output parameter. The growing trend of the line graph for each *x*-axis input/output parameter indicates a positive/negative connection with the *y*-axis input/output parameter under consideration. The straight line, on the other hand, demonstrates no link between the parameters. The correlation pattern of input parameters with the CS is depicted in Figure 2.

### 2.2. Machine Learning Algorithms Employed

**Figure 3:**
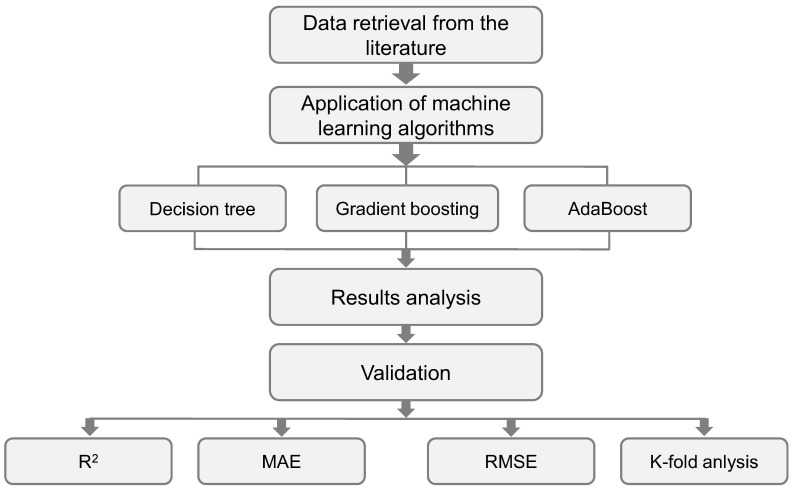
Flowchart of research methodology.

In order to meet the study′s aims, an individual ML method (SVM) and ensemble ML approaches (GB and XGB) were employed in conjunction with Python coding using the Anaconda Navigator package. Spyder (version 4.3.5) was used to run the SVM, GB, and XGB models. These techniques are frequently used to forecast desired outcomes in the presence of input parameters. These techniques can anticipate the temperature impacts, the strength characteristics, and the material′s durability, among other things [74,75]. Nine input factors and one output (CS) were used throughout the modeling phase. The projected result’s R^2^ value reflects the performance of the models employed. The R^2^ value indicates the degree of divergence; a value near zero indicates greater divergence, while a value near one indicates that the model and experimental data are nearly perfectly fit [40]. The subsequent sub-segments describe the ML approaches used in this study. Moreover, k-fold, statistical, as well as error evaluations were performed on all models involving root mean square error (RMSE) and mean absolute error (MAE). Additionally, a model-independent post hoc procedure called SHapley Additive exPlanations (SHAP) was used to examine the impact of input factors on the CS of GPCs. The research plan is depicted in Figure 3.

#### 2.2.1. Support Vector Machine

**Figure 4:**
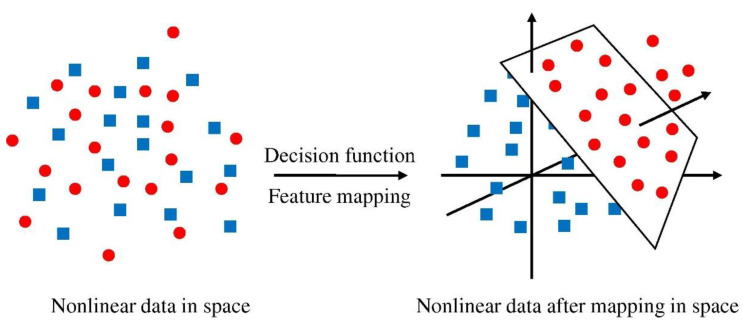
Support vector machine model mapping [76].

**Figure 5:**
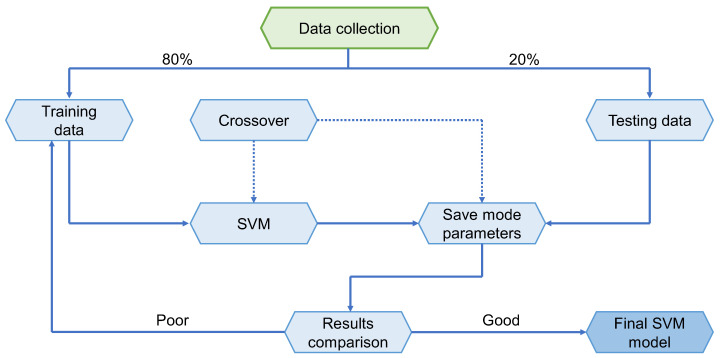
Support vector machine algorithm schematic representation.

SVM is a term that refers to supervised learning algorithms and related learning algorithms employed to evaluate data for classification and regression evaluation. An SVM technique is a description of the samples as points in space that have been plotted in such a manner that the patterns of the distinct classifications are split by a distinct vector (line/plane) with a gap as large as feasible. Other instances are then overlaid into that similar space and classified according to which side of the vector they lie on, as seen in Figure 4. Figure 5 depicts the SVM model’s implementation method. The material strength was estimated using this model, which takes into consideration the combined influence of several elements. The optimization technique was utilized to ascertain the SVM model’s parameters.

#### 2.2.2. Gradient Boosting

**Figure 6:**
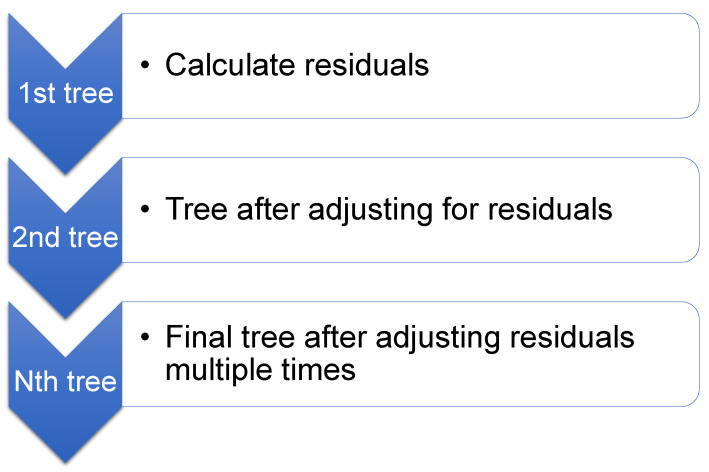
Schematic representation of gradient boosting technique [79].

In 1999, Friedman [77] proposed GB as a classification and regression ensemble approach. GB is useful exclusively for regression. Figure 6 depicts that the GB method relates every single repetition of the arbitrarily selected training dataset to the base model. By arbitrarily subsampling the training dataset, which also prevents overfitting, execution time may be sped up, and accuracy can be raised. The less the amount of training dataset, the quicker the regression since each iteration of the model must include minimal data. GB technique involves modification parameters, comprising *n*-trees and shrinkage rate, where *n*-trees are the number of trees to be formed; *n*-trees must not be maintained too small, and the shrinkage factor, also known as the learning rate, applied to all trees in progress, must not be kept too high [78].

#### 2.2.3. Extreme Gradient Boosting

**Figure 7:**
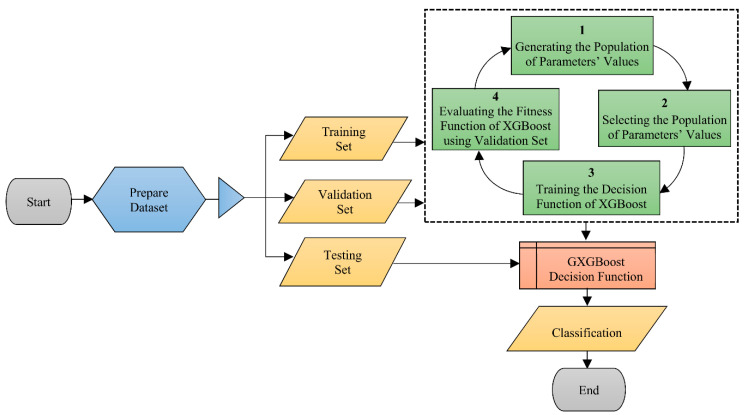
Schematic representation of extreme gradient boosting technique [80].

The fundamental concept underlying the projected XGB model is to construct an optimization job utilizing a genetic algorithm on top of the classifier in order to improve the classification precision of smaller groups, devoid of substantially compromising the classification precision of other groups. The genetic algorithm creates arbitrary estimates for the XGB in order to establish a new decision threshold with the greatest genetic fitness rate [80]. Particularly, the XGB model consists of the following four phases: producing the population of parameter values, choosing the population of parameter values, training the decision function, and assessing the fitness function. Figure 7 depicts the XGB flowchart.

## 3. Analysis of Results

### 3.1. Support Vector Machine Model

**Figure 8:**
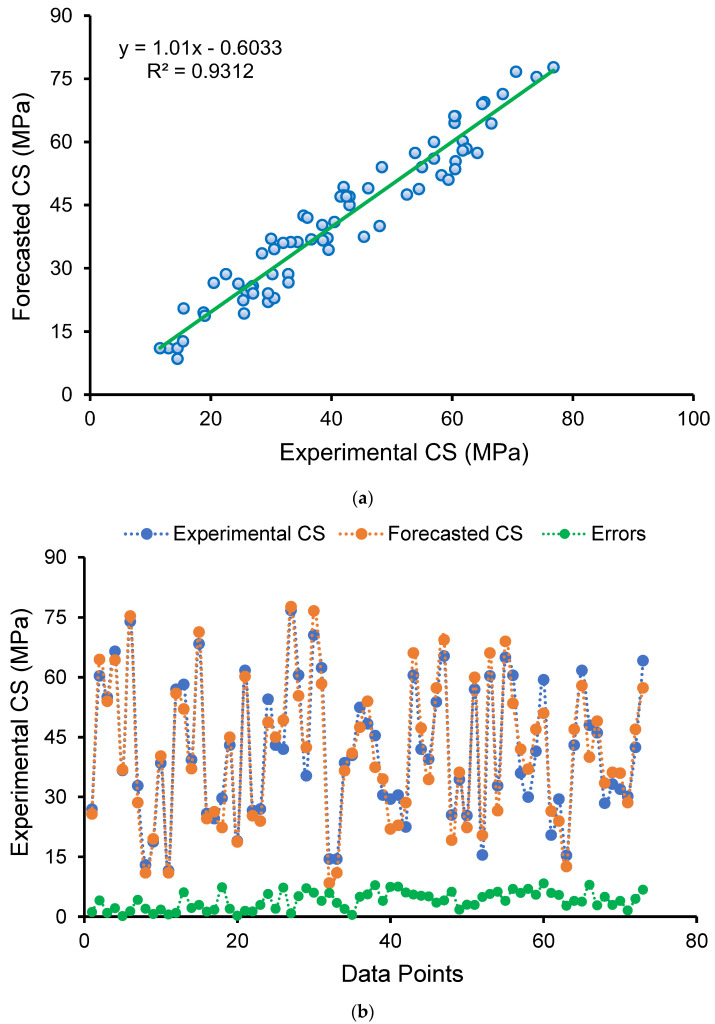
Support vector machine model: (**a**) connection amongst actual and forecasted findings; (**b**) scattering of experimental and estimated results.

Figure 8 depicts the findings of the SVM method for GPC’s CS. Figure 8a illustrates the relationship between actual data (experimental) and forecasted results. The SVM method yielded outcomes with a fair degree of precision and a minor discrepancy between actual and forecasted findings. The R^2^ value of 0.93 demonstrates the higher accuracy of the SVM technique in predicting the CS of GPC. Figure 8b depicts the scattering of experimental, projected, and divergence values (errors) for the SVM model. After analyzing the error values, it was discovered that the minimum, mean, and maximum values were 0.20 MPa, 4.04 MPa, and 8.39 MPa, respectively. In addition, the proportion dissemination of divergence values was established, and it was observed that 37% were lying below 3 MPa, 37% were in the range of 3–6 MPa, and 26% were greater than 6 MPa. In addition, the variance of diverged values suggests that the SVM technique performed adequately in predicting the CS of GPC.

### 3.2. Gradient Boosting Model

**Figure 9:**
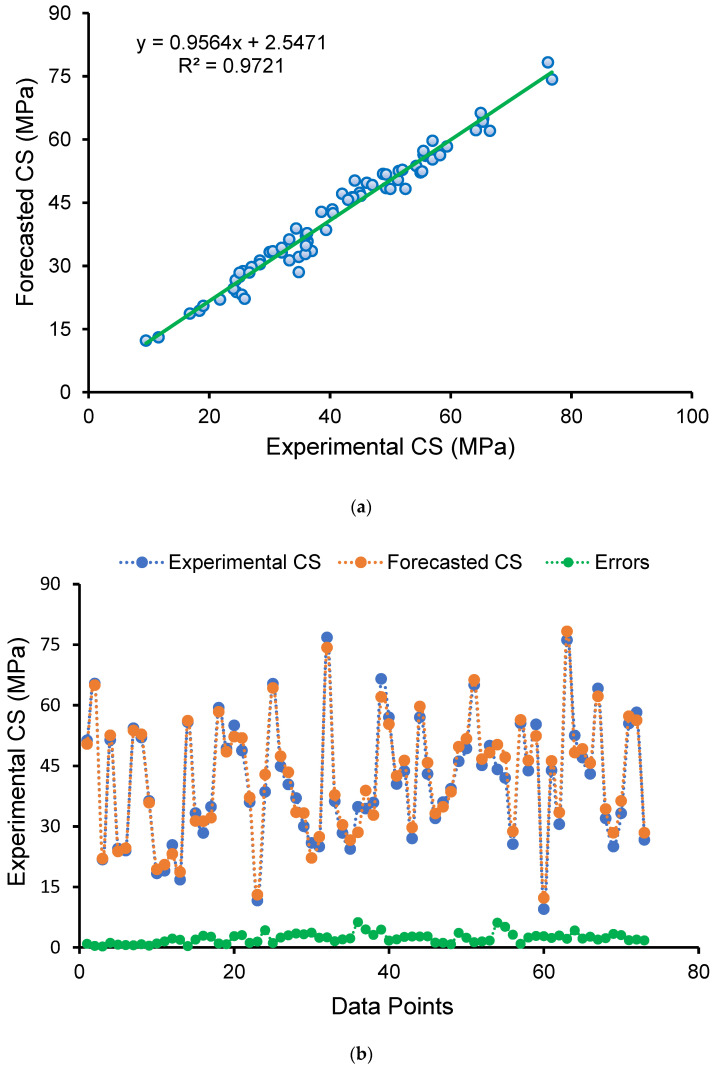
Gradient boosting model: (**a**) connection amongst actual and forecasted findings; (**b**) scattering of experimental and estimated results.

Figure 9a,b present an evaluation of the actual and estimated findings of the GB model. Figure 9a illustrates the correlation between actual and forecasted results, with an R^2^ of 0.97 indicating that the GB technique is more accurate than the SVM in forecasting the CS of GPC. The scattering of experimental, projected, and diverged values (errors) for the GB technique are depicted in Figure 9b. It was determined that the lowest, average, and maximum errors were 0.23 MPa, 2.27 MPa, and 6.30 MPa, respectively. The error distribution was 16.4% below 1 MPa, 60.3% between 1 and 3 MPa, and 23.3% above 3 MPa. In addition, these decreased error levels suggest that the GB model is more accurate than the SVM model. The improved accuracy of the GB model is due to the formation of twenty sub-models and using the one with the optimized R^2^ value.

### 3.3. Extreme Gradient Boosting Model

**Figure 10:**
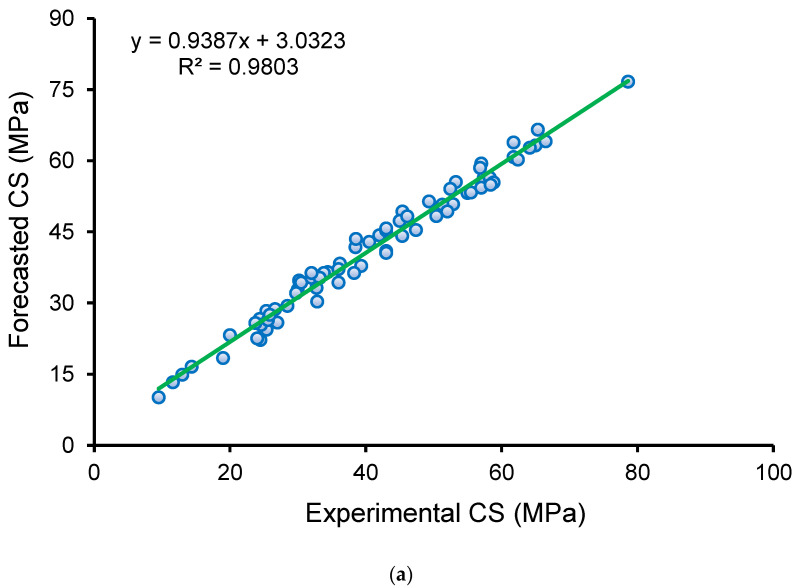

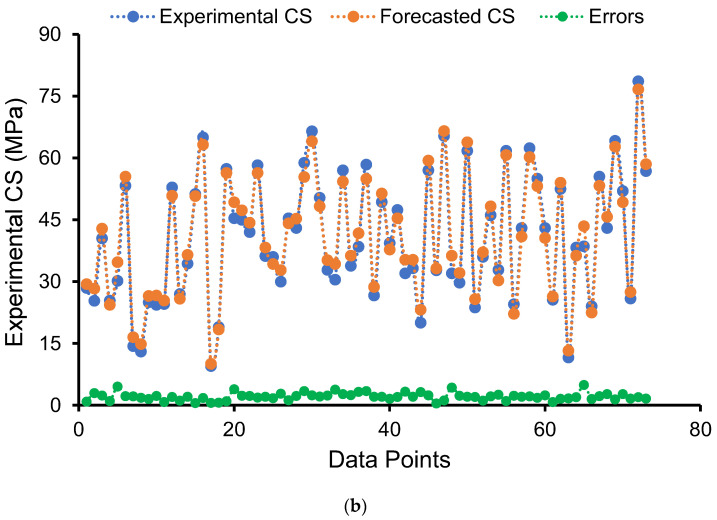
XGB model: (**a**) connection amongst actual and forecasted results; (**b**) scattering of experimental and estimated results.

Figure 10 displays the outcomes of the XGB method for predicting the GPC’s CS. Figure 10a depicts the connection between actual and forecasted findings. The XGB method yielded output with the best precision and the smallest deviation between experimental and predicted results. The XGB model is highly good at predicting the CS of GPC, with an R^2^ of 0.98. The scattering of experimental, forecasted, and diverged values (errors) for the XGB method is depicted in Figure 10b. The minimum, average, and highest error values were analyzed to be 0.44 MPa, 2.08 MPa, and 4.98 MPa, respectively. The error division was 10.0% below 1 MPa, 75.5% between 1 and 3 MPa, and 14.5% above 3 MPa. In addition, the division of errors reveals that the XGB model has the best predicting precision.

## 4. Models’ Validation

**Figure 11:**
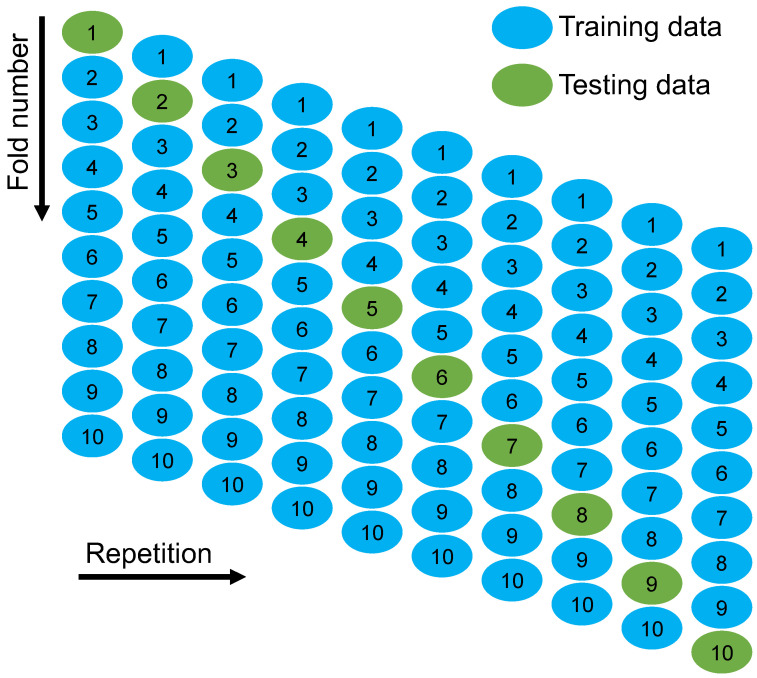
K-fold cross-validation procedure [84].

Methods of k-fold and statistical tests were utilized to validate the employed ML methods. Frequently, the k-fold approach [81] was used to determine the validity of a strategy by arbitrarily scattering and dividing pertinent data into 10 groups. As displayed in Figure 11, nine groups were utilized for training ML models, whereas one was to validate it. When the errors (MAE and RMSE) are small, and R^2^ is superior, the ML method is more precise. Moreover, the procedure must be repeated 10 times in order to obtain a satisfactory result. This substantial effort considerably adds to the exceptional accuracy of the model. In addition, as indicated in Table 2, each model was statistically tested for inaccuracy (MAE and RMSE). The MAE values for SVM, GB, and XGB were determined to be 4.03, 2.26, and 2.01 MPa, respectively. Similarly, the RMSE values for SVM, GB, and XGB were identified as 4.62, 2.59, and 2.18 MPa, respectively. These evaluations also suggested that the XGB model is more accurate than the other techniques because of its reduced errors. By using Equations (1) and (2), which were acquired from earlier studies [82,83], the prediction performance of the approaches was measured statistically.

(1) $$\mathrm{MAE}=\frac{1}{n}\sum_{i=1}^{n}\left|P_{i}-Ti\right|,$$

(2) $$\mathrm{RMSE}=\sqrt{\sum \frac{{\left(P_{i}-T_{i}\right)}^{2}}{n}},$$

where *n* = total number of data samples, $P_{i}\;$= forecasted values, and $T_{i}$ = actual values from the data sample.

**Figure 12:**
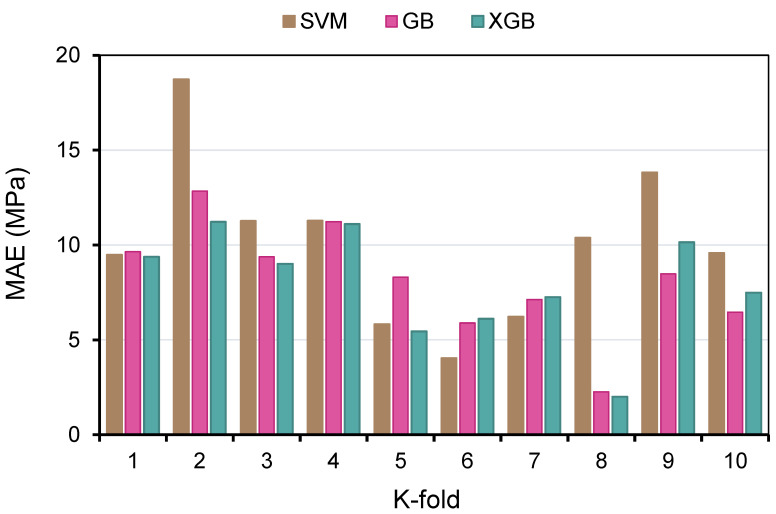
Mean absolute error distribution from k-fold analysis.

**Figure 13:**
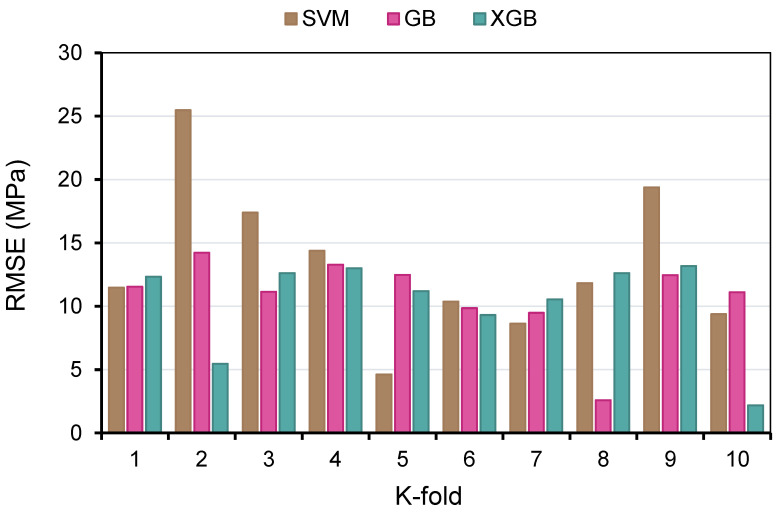
Root mean square error distribution from k-fold analysis.

**Figure 14:**
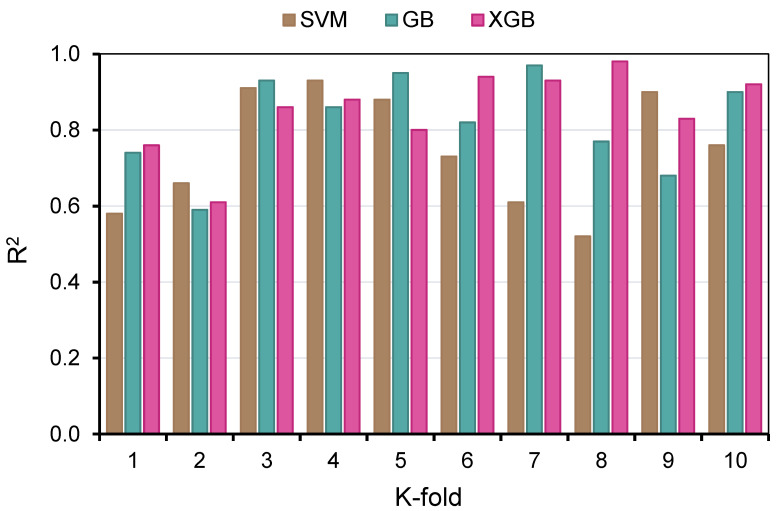
Coefficient of determination (R^2^) distribution from the k-fold analysis.

In order to determine the efficacy of the k-fold assessment, R^2^, RMSE, and MAE were computed, and their readings are listed in Table 3. In order to compare the outcomes of k-fold analysis for each ML method, Figure 12, Figure 13 and Figure 14 were constructed. As shown in Figure 12, the MAE values of the SVM technique varied from 4.03 to 18.73 MPa with an average of 10.06 MPa. The MAE readings for the GB model ranged from 2.26 to 12.84 MPa, with an average of 8.16 MPa. Moreover, the MAE readings for the XGB technique varied between 2.01 and 11.22 MPa, with an average of 7.92 MPa. Similarly, the average RMSE readings for the SVM, GB, and XGB models are 13.29, 10.81, and 10.24 MPa, respectively (Figure 13). However, the average R^2^ for SVM, GB, and XGB were 0.75, 0.82, and 0.85, respectively (Figure 14). Compared to the other models, the XGB model with the lowermost error values and the greatest R^2^ is the most exact in forecasting the CS of GPC.

## 5. Influence of Input Parameters

**Figure 15:**
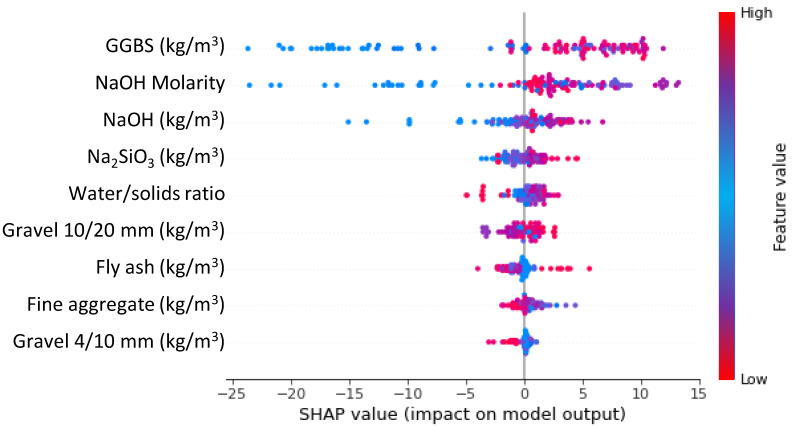
SHAP plot indicating the influence of input parameters on ML models.

In this study, the impact of input factors on the ML technique′s performance was determined. SHAP tree explainer is originally applied over the whole database in order to offer a more accurate description of global feature impacts by combining local SHAP explanations. The “TreeExplainer” technique of tree-like SHAP approximation was implemented [85]. This approach evaluates the internal structure of tree-based models, i.e., the summation of a series of computations associated with the tree model′s leaf node, which leads to low-order complexity. Figure 15 displays the results on the violin SHAP plot for all the characteristics used to predict the CS of GPC. In this plot, each feature value is represented by a distinct color, and the corresponding SHAP value on the *x*-axis indicates the contribution output. As an example, GGBS is an input characteristic with a greater impact, illustrating the greater positive link between this characteristic and the CS of GPC. This indicates that an increase in GGBS would mostly lead to an increase in CS. A comparable impact of NaOH molarity, NaOH, and Na_2_SiO_3_ was also indicated on the CS prediction of GPC. The water/solids ratio, Gravel 10/10 mm, and fly ash have both beneficial and negative effects on the GPC’s CS. This signifies that using these ingredients up to optimal contents improves the CS, while at higher contents, the CS of GPC decreases. On the other hand, the fine aggregate and gravel size, 4/10 mm, have a less positive influence and more negative influence (more red dots on the negative side). This evaluation is based on a database suggested by current research, and more data points may yield more accurate results.

**Figure 16:**
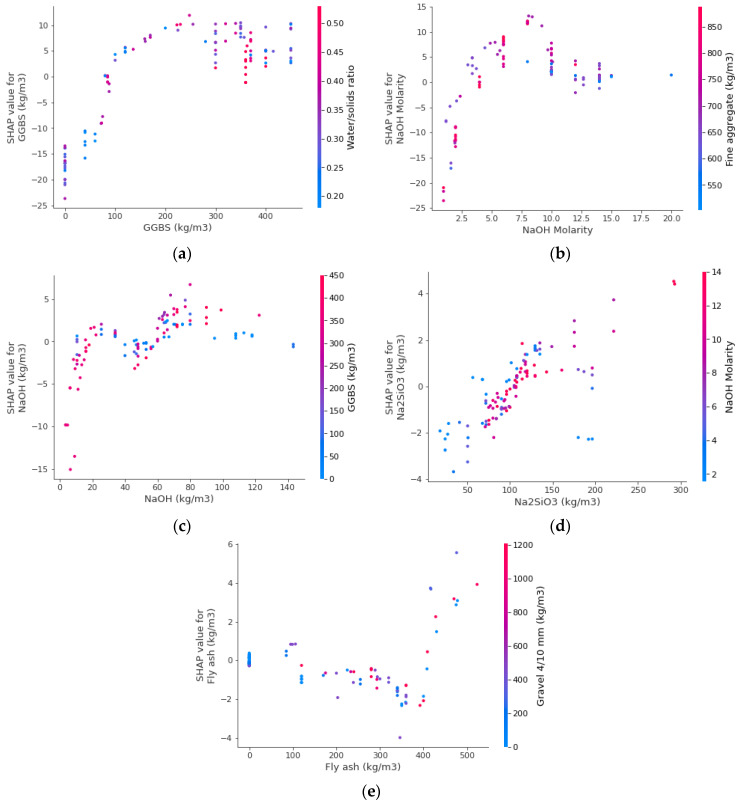
Interaction plots: (**a**) GGBS; (**b**) NaOH molarity; (**c**) NaOH; (**d**) Na_2_SiO_3_; (**e**) fly ash.

Figure 16 depicts the relationship between the input parameters and the GPC’s CS. Figure 16a depicts the interaction of the GGBS. The scatter plot reveals that, among other parameters, GGBS has the highest impact on the CS of GPC, which is increasing with increasing GGBS quantity and is mainly interacting with the water/solids ratio. By increasing the GGBS quantity from 0 to 300 kg/m^3^, the CS of GPC improves incessantly, while above that value, its impact becomes constant. In this circumstance, the quantity of GGBS in the range of 300–400 kg/m^3^ is favorable in achieving high CS for GPCs, while using the same ingredients considered in the present study. Similarly, NaOH molarity, NaOH, and Na_2_SiO_3_ have a favorable impact on the CS of GPC with increasing amounts up to an optimal content (Figure 16b–d). For example, increasing NaOH molarity up to eight has a positive influence, while further increase causes a negative impact (Figure 16b). However, the impact of fly ash on the CS of GPC is different. Using fly ash below 400 kg/m^3^ has a detrimental influence while using fly ash in higher amounts has a favorable impact on the CS of GPC. It is important to mention here that these observations are based on the types of ingredients and the number of data samples considered in this study. Using distinct ingredients as input parameters and datasets might yield different outputs.

## 6. Discussion

**Figure 17:**
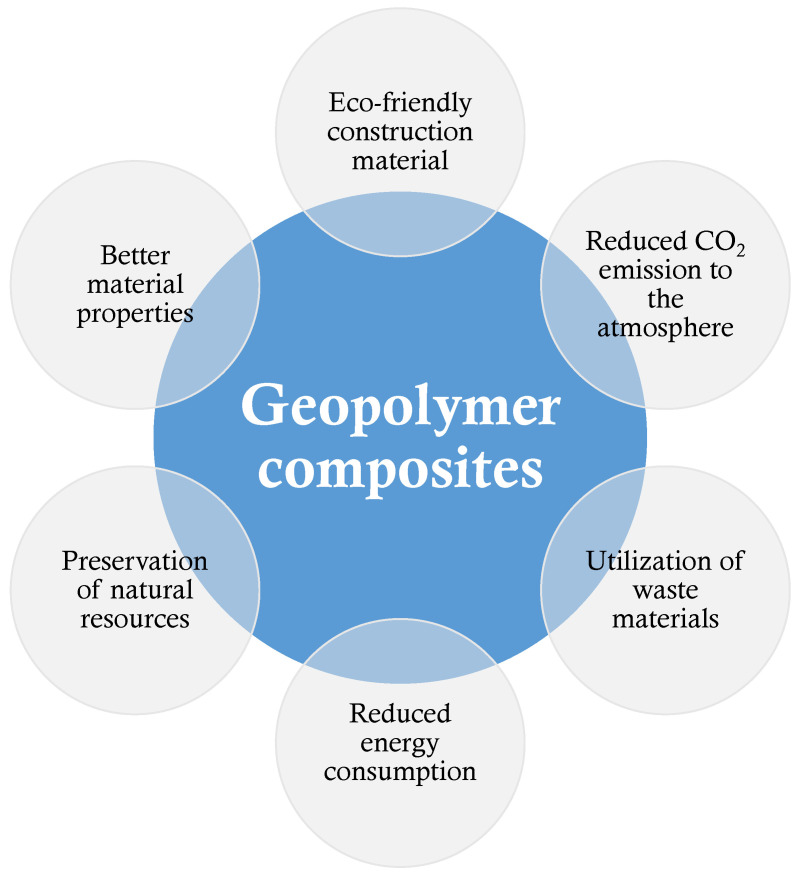
Pros of geopolymers manufactured with waste materials.

**Figure 18:**
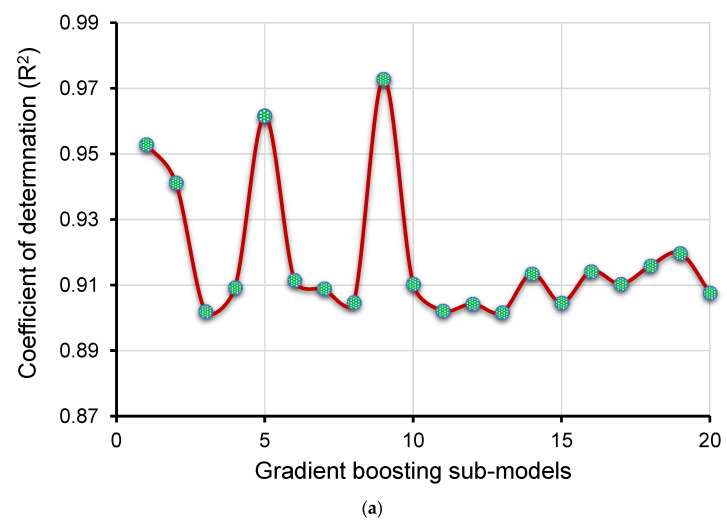

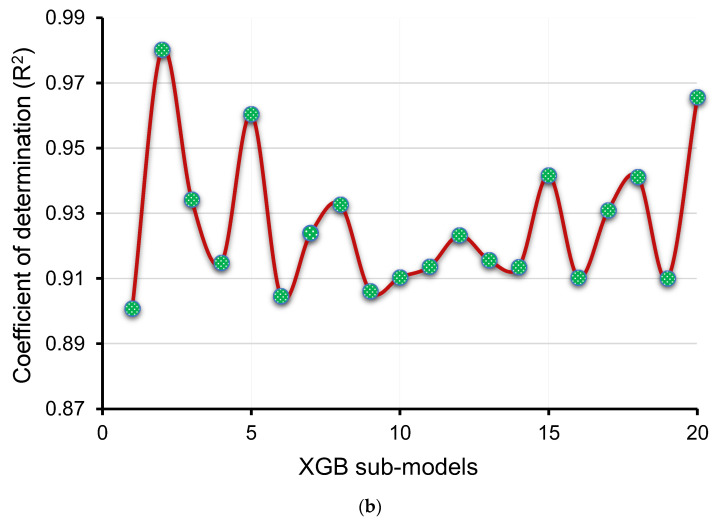
R^2^ of sub-models: (**a**) gradient boosting; (**b**) XGB.

The purpose of this research was to contribute to the current body of knowledge concerning the application of modern methods for measuring the CS of GPC. This study will benefit the construction industry by providing quick and cost-effective methods for predicting material properties. In addition, the acceptance and usage of GPC in the building sector will be hastened by employing these measures to encourage eco-friendly building. As GPC might be produced from aluminosilicate-containing wastes, its usage in the building sector will offer many benefits, as shown in Figure 17. This research illustrates how ML techniques may be utilized to predict the CS of GPC. The study employed three ML methods: one individual (SVM) and two ensembled (GB and XGB). The accuracy of each approach was evaluated to determine the highly effective forecaster. In comparison with the GB and SVM techniques, which provided R^2^ of 0.97 and 0.93, respectively, the XGB technique produced a more accurate result with an R^2^ of 0.98. In comparison, Wang et al. [84] also anticipated the CS of geopolymer concrete by using the AdaBoost, random forest, and decision tree algorithms and reported the R^2^ value equal to 0.90, 0.90, and 0.83, respectively. Cao et al. [86] also employed SVM and MLP approaches for the CS of geopolymer concrete and reported the R^2^ result as 0.91 and 0.88, respectively. It also indicates that the selected algorithms in the present study performed better than the approaches used in the previous studies. In addition, the correctness of each model was tested using the k-fold and statistical methods. The greater the model’s precision, the fewer errors it contains. Nevertheless, establishing and suggesting the ideal ML method for anticipating findings in a range of domains is challenging since the performance of an ML method is highly dependent on the quantity of input variables dataset used to run algorithms. In contrast, ensembled ML techniques frequently utilize the weak learner by building sub-models that might be trained on the dataset and optimized to maximize the R^2^. Figure 18 depicts the distribution of R^2^ for the GB and XGB submodels. Figure 18a depicts the R^2^ values for GB sub-models with the lowest, mean, and maximum R^2^ values of 0.902, 0.919, and 0.973, respectively. Nonetheless, the lowest, mean, and maximum R^2^ values for XGB submodels were 0.901, 0.927, and 0.980, respectively (Figure 18b). These results indicate that both the GB and XGB submodels have similar readings and a superior degree of accuracy when predicting the GPC’s CS. Additionally, SHAP was used to investigate the effect of input factors on the CS of GPCs. GGBS was determined to be an input feature with a bigger influence, indicating a stronger positive relationship between this characteristic and GPC’s CS. On the CS prediction of GPC, a comparable effect of NaOH molarity, NaOH, and Na_2_SiO_3_ was also revealed. Gravel size: 10/20 mm, the water/solids ratio, and fly ash have both positive and negative influences on the GPC’s CS. This indicates that employing these substances up to the ideal concentration enhances the CS, but the CS of GPC diminishes at greater concentrations. In contrast, the fine aggregate and gravel size of 4/10 mm have a lesser beneficial impact and a greater negative impact.

## 7. Conclusions

This research aimed to utilize both individual and ensemble machine learning (ML) methods to predict the compressive strength (CS) of geopolymer composites (GPCs). One individual approach, support vector machine (SVM), and two ensemble strategies, gradient boosting (GB) and extreme gradient boosting (XGB), were employed to predict outcomes. As a consequence of this investigation, the following findings were drawn:

- Ensemble ML methods fared better at predicting the CS of GPCs than individual machine learning techniques, with the XGB model doing the best. For the XGB, GB, and SVM models, the coefficients of determination (R^2^) were 0.98, 0.97, and 0.93, respectively. All the employed techniques yielded results within a satisfactory limit and with little deviation from the experimental findings;
- These error readings also proved the best performance of the XGB method in forecasting the CS of GPC;
- Statistical tests and k-fold analysis validated the performance of the employed models. The smaller errors and greater R^2^ resulting from k-fold analysis suggested the higher precision of the ML model. These analyses indicated that the XGB model outperformed the other investigated models;
- Based on the results of the SHAP analysis, GGBS was considered to be a more influential input feature, showing a larger positive association between this characteristic and GPC’s CS;
- This nature of research will help the construction industry by facilitating the development of fast and cost-efficient methods for forecasting material characteristics. Moreover, by supporting eco-friendly construction, these initiatives will hasten the acceptance and application of GPC in the building sector.

Further studies can also include the other experimental factors, such as the geometry of the sample testing, strain rate, and temperature effect. The other ensemble ML approaches can also be employed to check the precision level of the results, such as random forest, bagging, and boosting.

## Figures and Tables

**Table 1:** Descriptive measurements of input and output variables.

Parameter	Fine Aggregate (kg/m^3^)	GGBS (kg/m^3^)	Fly Ash (kg/m^3^)	NaOH Molarity	Water/Solids Ratio	Na_2_SiO_3_ (kg/m^3^)	NaOH (kg/m^3^)	Gravel Size: 4/10 mm (kg/m^3^)	Gravel Size: 10/20 mm (kg/m^3^)	CS (MPa)
Mean	729.88	225.15	174.34	8.14	0.34	111.66	53.74	288.39	737.37	43.28
Mode	651	0	0	10	0.53	108	64	0	0	56.00
Median	728	300	120	9.2	0.34	108	56	208	789	42.10
Maximum	1360	450	523	20	0.63	342	147	1293.4	1298	86.08
Minimum	459	0	0	1	0	18	3.5	0	0	8.00
Standard Deviation	130.97	162.27	167.95	4.56	0.11	48.16	31.91	372.31	358.55	17.87
Sum	26,4947.79	81,728.05	63,286.04	2955.11	124.78	40,532.68	19,508.75	104,684.28	267,664.93	15,710.40
Range	901	450	523	19	0.63	324	143.5	1293.4	1298	78.08
Standard Error	6.87	8.52	8.82	0.24	0.01	2.53	1.67	19.54	18.82	0.94

**Table 2:** Statistical assessments of the ML methods employed.

Machine Learning Model	MAE (MPa)	RMSE (MPa)
Support vector machine	4.03	4.62
Gradient boosting	2.26	2.59
Extreme gradient boosting	2.01	2.18

**Table 3:** Results of k-fold evaluation.

K-Fold	Support Vector Machine			Gradient Boosting			Extreme Gradient Boosting		
	MAE (MPa)	RMSE (MPa)	R^2^	MAE (MPa)	RMSE (MPa)	R^2^	MAE (MPa)	RMSE (MPa)	R^2^
1	9.48	11.47	0.58	9.64	11.54	0.74	9.38	12.33	0.76
2	18.73	25.48	0.66	12.84	14.23	0.59	11.22	5.45	0.61
3	11.27	17.38	0.91	9.38	11.14	0.93	9.00	12.61	0.86
4	11.28	14.38	0.93	11.23	13.28	0.86	11.10	13.00	0.88
5	5.82	4.62	0.88	8.30	12.48	0.95	5.45	11.19	0.80
6	4.03	10.38	0.73	5.89	9.84	0.82	6.11	9.30	0.94
7	6.22	8.63	0.61	7.12	9.48	0.97	7.25	10.55	0.93
8	10.38	11.83	0.52	2.26	2.59	0.77	2.01	12.61	0.98
9	13.82	19.38	0.90	8.48	12.45	0.68	10.15	13.18	0.83
10	9.58	9.37	0.76	6.46	11.10	0.90	7.49	2.18	0.92

## Data Availability

The data used in this research have been properly cited and reported in the main text.

## Supplementary Materials

The following supporting information can be downloaded at: https://www.mdpi.com/article/10.3390/polym14122509/s1, Table S1: Data used for modeling.
